# Using online public animal price data as a signal for predicting an increase in animal disease outbreak reports: a pilot study on cross-correlation modeling in Thailand

**DOI:** 10.1186/s12917-025-04888-5

**Published:** 2025-07-02

**Authors:** Veerasak Punyapornwithaya, Supitchaya Srisawang, Chalita Jainonthee, Wengui Li, Ronello Abila, Bolortuya Purevsuren

**Affiliations:** 1 Research Center of Veterinary Biosciences and Veterinary Public Health, Faculty of Veterinary Medicine, Chiang Mai University, Chiang Mai, 50100 Thailand; 2 College of Veterinary Medicine, Yunnan Agricultural University, Kunming, 650201 China; 3 World Organisation for Animal Health (WOAH) Sub-Regional Representation for South East Asia, Bangkok, 10400 Thailand

**Keywords:** Animal price, Transboundary animal disease, Outbreak, Cross-correlation, Time lag, Signal, Online public data

## Abstract

**Background:**

Changes in livestock prices are often linked to disease outbreaks. An animal price monitoring system has been considered a potential tool for predicting transboundary animal diseases (TADs). The aim of this study was to examine the cross-correlation between market price dynamics and disease outbreak patterns using publicly available online data to explore the potential of market prices as early indicators of impending TAD outbreaks.

**Methods:**

Time series data on TAD outbreak reports, including foot and mouth disease (FMD), lumpy skin disease (LSD), and African swine fever (ASF), as well as animal price data for cattle and pigs in Thailand, were analyzed. Cross-correlation analysis was conducted to assess the relationship between animal prices and disease outbreak report patterns. Data from January 2021 to December 2023 (primary dataset) were analyzed to identify cross-correlation patterns, while data from January to September 2024 (extended dataset) were incorporated to evaluate the consistency of the observed cross-correlation over the study period.

**Results:**

A significant cross-correlation was identified between cattle prices and the number of outbreak reports for FMD in the primary dataset. An increase in cattle prices during the preceding one to two months (lags of -1 and − 2) was associated with a subsequent rise in FMD outbreak reports. This correlation remained consistent when the extended dataset was incrementally incorporated and analyzed on a month-by-month basis. In contrast, in the primary dataset, no significant cross-correlation was observed between cattle prices and LSD outbreak reports. For ASF, cross-correlations between farm-gate pig prices and ASF outbreak reports were detected at lag 0, lag 3, lag 4, and lag 5 in the primary dataset; however, no significant correlation was observed in the extended dataset.

**Conclusions:**

This study demonstrates the feasibility of using animal price trends as signal tools for anticipating an increase in TAD outbreak reports. The findings specifically support the use of cattle price data as an early signal for forecasting increases in FMD outbreak reports in Thailand. The availability and consistency of publicly accessible data are essential components for the feasibility of using animal prices as a signal tool. Decision-makers and veterinary authorities may incorporate such tools into surveillance systems to support early warning efforts.

**Supplementary Information:**

The online version contains supplementary material available at 10.1186/s12917-025-04888-5.

## Background

Numerous countries in Asia face significant threats from transboundary animal diseases (TADs) including foot and mouth disease (FMD), lumpy skin disease (LSD) and African swine fever (ASF). FMD remains endemic in several countries across the region posing ongoing negative impacts on livestock health and trade [1–4]. LSD, first identified in South Asia in 2019, has spread to East and Southeast Asia (SEA), with outbreaks being continuously reported to the World Organisation for Animal Health (WOAH) [5, 6]. Similarly, ASF outbreaks have caused severe economic losses in several Asian countries [7–14].

Previous studies have demonstrated an association between the trade of live animals and the spread of FMD and LSD [15, 16]. Moreover, fluctuations in livestock prices are often associated with disease outbreaks and their impact on animal populations. For example, during the ASF outbreak in Thailand, farm-gate pig prices rose sharply [17]. Similarly, ASF outbreaks in China and other affected countries resulted in significant increases in farm-gate pig prices [18–20]. Additionally, a study in Korea indicated that ASF news had an impact on the price of pork [21]. While the relationship between animal disease outbreaks and livestock prices has been well-documented, research exploring the use of animal prices as early warning signals for disease outbreaks remains limited.

Monitoring fluctuations in animal prices as a potential method for predicting TADs has garnered interest from international animal health organizations. In response, a pilot study was conducted to evaluate the feasibility of establishing an Animal Price Monitoring System (APMS) to predict TADs in Asia [22]. However, the study faced several challenges, primarily the lack of essential data, leading to the conclusion that establishing an APMS at the regional level was not feasible at that time. To address these challenges, the focus of the study was scaled down to the national level. Thailand was selected for this pilot study due to the availability of relevant publicly accessible data. The country operates an established system for managing animal disease outbreaks, with the Department of Livestock Development (DLD) serving as the primary authority responsible for disease control and surveillance. For TADs such as FMD, ASF, and LSD, the DLD follows standardized procedures that typically include outbreak investigation, sample collection, laboratory confirmation, and reporting from local to central levels. Once an outbreak is confirmed, the DLD implements corresponding control and surveillance measures to mitigate disease spread [23–25].

Animal prices and outbreak data collected at regular intervals (e.g., daily, weekly, or monthly), are categorized as time series data [26]. The relationship between two time series can be assessed using cross-correlation analysis [27–29], enabling the determination of correlations between animal prices and outbreak occurrences. Given that cross-correlation analysis provides valuable insights into whether changes in one variable precede changes in another, it could, therefore, serve as an effective warning signal for the other variable.

This study explored the cross-correlation between animal prices and three important TADs in Thailand including FMD, LSD, and ASF. FMD was included as it is an endemic livestock disease in the country [23]. As such, FMD serves as a representative example of an endemic disease with consistent outbreak patterns over time. LSD was selected for its contrasting epidemiological profile. First reported in Thailand in 2021, LSD experienced a high number of outbreak reports for a few months before declining to consistently low or absent levels [30, 31]. Similarly, ASF exhibited a short-lived peak in outbreak reports, followed by an extended period with no reported outbreaks. Both LSD and ASF serve as representatives of diseases characterized by a short outbreak peak followed by minimal or no subsequent reports. Furthermore, in terms of animal price dynamics, ASF serves as a representative example of a situation in which the government implemented intervention policies to impose a price ceiling on pigs during the ASF epidemic.

To our knowledge, studies exploring the use of statistical models to apply animal prices as a signal for predicting TADs are very limited, resulting in a significant knowledge gap on this topic. Therefore, the objective of this study was to investigate the cross-correlation between animal price dynamics and disease outbreak patterns, utilizing publicly available online data to assess the potential of animal prices as early indicators of forthcoming outbreaks. Furthermore, in supporting the study’s aim, the cross-correlation between animal movement and outbreak reports was also investigated to gain a better understanding of their relationships. The findings from this pilot study would serve as a baseline source of information if the establishment of an APMS for early outbreak detection is intended.

## Methods

### Data and definitions

This study utilized publicly available online animal price data from the Department of Livestock Development (DLD) (https://dld.go.th) and the Office of Agricultural Economics (https://www.oae.go.th) in Thailand. Data on animal disease outbreaks, including FMD, LSD, and ASF, were sourced from the World Animal Health Information System (WAHIS) (https://wahis.woah.org). Additionally, animal movement data were obtained from the Division of Veterinary Inspection and Quarantine, DLD (https://aqi.dld.go.th/webnew/index.php/th).

In this study, outbreak reports refer to the number of recorded outbreak events for each disease, including FMD, LSD, and ASF, aggregated on a monthly basis from January 2021 to September 2024. Each recorded outbreak event includes information on the geographic location of the outbreak, represented by the centroid of the outbreak area, along with subdistrict and higher administrative unit data.

Monthly animal prices and outbreak data were categorized into two datasets: the primary dataset, spanning January 2021 to December 2023, and the extended dataset, covering January 2024 to September 2024. The primary dataset was employed to conduct the cross-correlation analysis, whereas the extended dataset was subsequently incorporated to validate the robustness and persistence of the observed associations upon the inclusion of additional temporal data.

### Cross-correlation analysis

Cross-correlation is a statistical technique applied to quantify the degree of similarity or association between two time series. The cross-correlation between two time series $\:x$ and $\:y$ at lag $\:\mathcal{l}$ is given by [32]:

$$\:{r}_{xy}\left(\mathcal{l}\right)={\sum\:}_{i=0}^{N-1}{x}_{i}{y}_{i}$$

where $\:{r}_{xy}\left(\mathcal{l}\right)$ is the cross-correlation at lag $\:\mathcal{l}$, which represents the number of data points by which the signal is shifted. The term $\:N$ is the number of data points in each data series. The term $\:{x}_{i}$ is the$\:{i}^{th}$ data point of the first time series and $\:{y}_{i}$ is the $\:{i}^{th}$ of the second time series.

In the cross-correlation analysis, $\:x$ can be set to precede $\:y$ or, alternatively, $\:y$ can precede $\:x$. When $\:x$ precedes $\:y$, it indicates that $\:x$ occurred earlier and may be associated with changes in $\:y$. In the present study, we set $\:x$ to precede $\:y$ for all cross-correlation analyses. To investigate its potential as a predictive signal, animal price was designated as the $\:x$ variable, while outbreak reports were assigned as the $\:y$ variable (Table 1; Models 1, 2 and 3).

**Table 1:** Details of cross-correlation models

Model	Variables (x γ y)*	Disease**	Species
1	Cattle prices **γ** outbreak reports	FMD	Cattle
2	Cattle prices **γ** outbreak reports	LSD	Cattle
3	Farm-gate pig prices **γ** outbreak reports	ASF	Pigs
4	Cattle prices **γ** number of FMD affected cattle	FMD	Cattle
5	Cattle movement reports **γ** outbreak reports	FMD	Cattle

*Cross-correlation is defined as the x-variable is considered to precede the-y variable

** FMD = foot and mouth disease, LSD = lumpy skin disease, ASF = African Swine Fever

Cross-correlation analysis was conducted using the $\:\text{c}\text{c}\text{f}\left(\right)$ function from the “*tseries*” package in R version 4.4.2 [33]. The results from the cross-correlation analysis include a table presenting the correlation coefficients for each monthly lag and graphs depicting bars for each lag, representing the corresponding correlation coefficient values. In the graphs, a bar exceeding the dotted line indicates a statistically significant cross-correlation between variables $\:x$ and $\:y$ at that specific lag.

The interpretation of cross-correlation results has been illustrated in a previous study, providing a framework for understanding the results of temporal relationships between time series data [28]. In brief, cross-correlation can be either positive or negative, and the associated time lag may also be positive or negative. For instance, a positive relationship with a negative time lag indicates that *x* precedes (occurs prior to) *y* by *t* time units. In this context, an increase in animal prices (*x*) precedes an increase in outbreak reports (*y*) by two months. In contrast, a positive relationship with a positive time lag indicates that *x* lags (occurs after) *y*, meaning that an increase in outbreak reports is followed by an increase in animal prices after *t *time units. Negative time lags are particularly relevant for identifying early signals, as they reflect situations where historical values of *x* (e.g., animal prices) are associated with future values of *y* (e.g., outbreak reports). For instance, a lag of -2 ($\:lag=-2)$ indicates that an increase in animal prices two months prior is correlated with a rise in outbreak reports in the current month.

Furthermore, if a significant cross-correlation between x and y was identified, extensions to the dataset were utilized to evaluate whether the predictive capability of the model remains consistent when updated data is incorporated. Accordingly, monthly data on animal prices and outbreak reports were progressively added in a stepwise manner. For example, data from January 2004 were initially included, and a cross-correlation analysis was performed. Subsequently, data from February 2004 were added to the dataset, which already included January 2004, and the analysis was repeated. This incremental process was continued month by month until the inclusion of data from September 2024.

Although not the primary objective of this study, additional analyses were conducted to provide deeper insights into the relationship between various factors and disease dynamics. In particular, two supplementary analyses were performed: (1) a cross-correlation analysis examining the relationship between cattle prices and the number of FMD affected cattle during outbreaks (Table 1; Model 4) and (2) a cross-correlation analysis exploring the connection between the number of cattle movement reports and the number of FMD outbreak reports (Table 1; Model 5). The models and additional details are summarized in Table 1.

### Generalized linear model

In cases where the cross-correlation between animal prices and outbreak reports was found to be statistically significant, a generalized linear model (GLM) with a negative binomial distribution (NB) was employed to quantify the relationship. This model determined the magnitude of change in outbreak reports corresponding to changes in animal prices.

### Baseline model

GLM with NB distribution was employed to assess the relationship between animal prices and the number of FMD outbreaks. This modeling approach was selected to account for overdispersion in the data, where the variance exceeds the mean. The model is represented as follows [34]:

$$\:{ln}({\lambda\:}_{i})={\beta\:}_{o}+{\beta\:}_{1}{x}_{1j}$$

The observed counts $\:{Y}_{i}\sim\:NB({\mu\:}_{i},\theta\:)$, where $\:{\mu\:}_{i}={\lambda\:}_{i}$ represents the expected mean number of outbreak reports, and $\:\theta\:$ is the dispersion parameter. The term$\:{x}_{1j}$ denotes the price at month $\:j$, corresponding to the furthest back in time prior to a change in outbreak reports, among the significant negative lags (*p* < 0.05) identified through cross-correlation analysis. For example, if significant cross-correlations are detected at multiple lags (e.g.,$\:j=-1$ and $\:j=-2$), the price lag $\:j=-2$ is selected for inclusion in the GLM analysis, as it provides the earliest possible signal for anticipating changes in outbreak reports.

### Seasonally adjusted model

To account for potential seasonal patterns in FMD outbreaks, a second NB model was fitted by incorporating calendar month as a categorical covariate:$\:{ln}({\lambda\:}_{i})={\beta\:}_{0}+{\beta\:}_{1}{x}_{1j}+{\beta\:}_{2}{x}_{2k}$The term $\:{x}_{2k}$ refers to the price at month $\:k$, included to account for calendar month effects through a set of monthly variables (e.g., January, February…, December).

Model performance was evaluated using the Akaike Information Criterion (AIC) to compare the relative fit between competing models. The model with the smaller AIC value was considered to provide a better fit to the data.

For model assumption evaluations, Pearson residuals were examined to assess the goodness of fit and identify any potential deviations from the model assumptions. Residuals were plotted against observation indices to detect patterns, heteroscedasticity, or outliers. Diagnostic plots of Pearson residuals were used to evaluate the goodness of fit of the negative binomial model. If the residuals appear randomly scattered around zero without discernible patterns, trends, or extreme outliers, the model assumptions can be considered reasonably satisfied. Under these criteria, the results derived from the model were considered valid for interpretation.

## Results

### Temporal trend of animal prices and outbreak reports

**Fig. 1:**
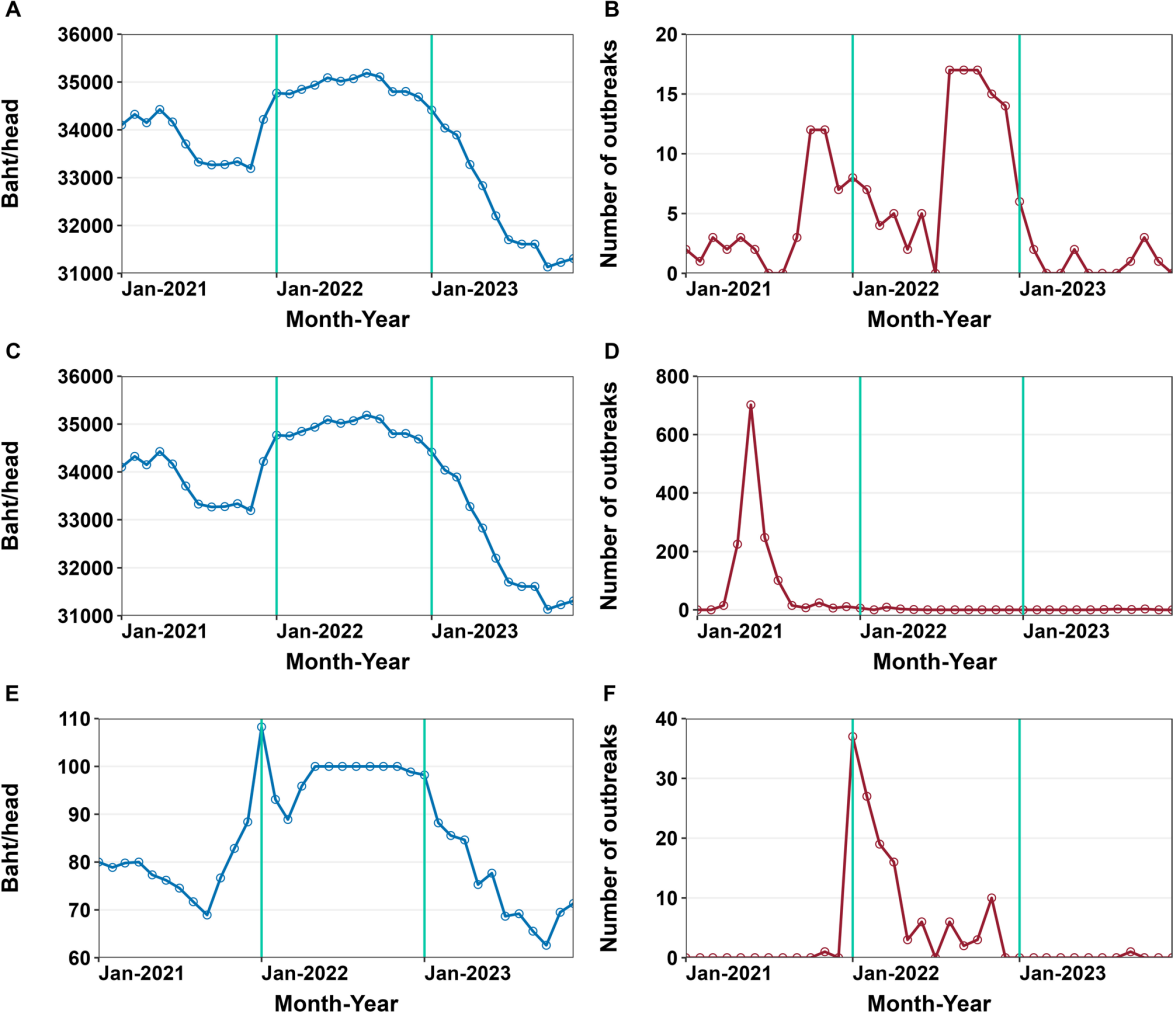
Temporal patterns of cattle prices (**A**) and foot and mouth disease outbreak reports (**B**), cattle prices (**C**) and lumpy skin disease outbreak reports (**D**), and farm-gate pig prices (**E**) and African swine fever outbreak reports (**F**)

In 2021, cattle prices fluctuated moderately from January to April, followed by a steady decline starting in May and a slight upward trend in November and December. In 2022, prices showed a slight but steady increase from January to September, then gradually declined from October 2022 through 2023 (Fig. 1A&C). In contrast, farm-gate pig prices increased from late 2021 to January 2022 and remained stable between May and October 2022 due to an intervention policy from the Thai government. However, from January to October 2023, farm-gate pig prices experienced a continuous decline (Fig. 1E).

FMD outbreak reports exhibited fluctuations over the study period. An initial increase was observed from September to December 2021, followed by a relatively stable period in early 2022. Outbreak reports rose again between August and December 2022. In 2023, the number of reported outbreaks decreased markedly, with multiple months recording no outbreaks. LSD outbreaks peaked between April and June 2023, followed by a sharp decline in July, with very few or no reported outbreaks from August 2023 to December 2023. Similarly, ASF outbreak reports reached a short peak in January 2022, after which the number of reported outbreaks declined. No outbreaks were reported from November 2022 to August 2023. In 2023, an outbreak was reported only in September. (Fig. 1).

### Cross-correlation analysis

#### Animal prices and outbreak reports (Models 1, 2, and 3)

**Fig. 2:**
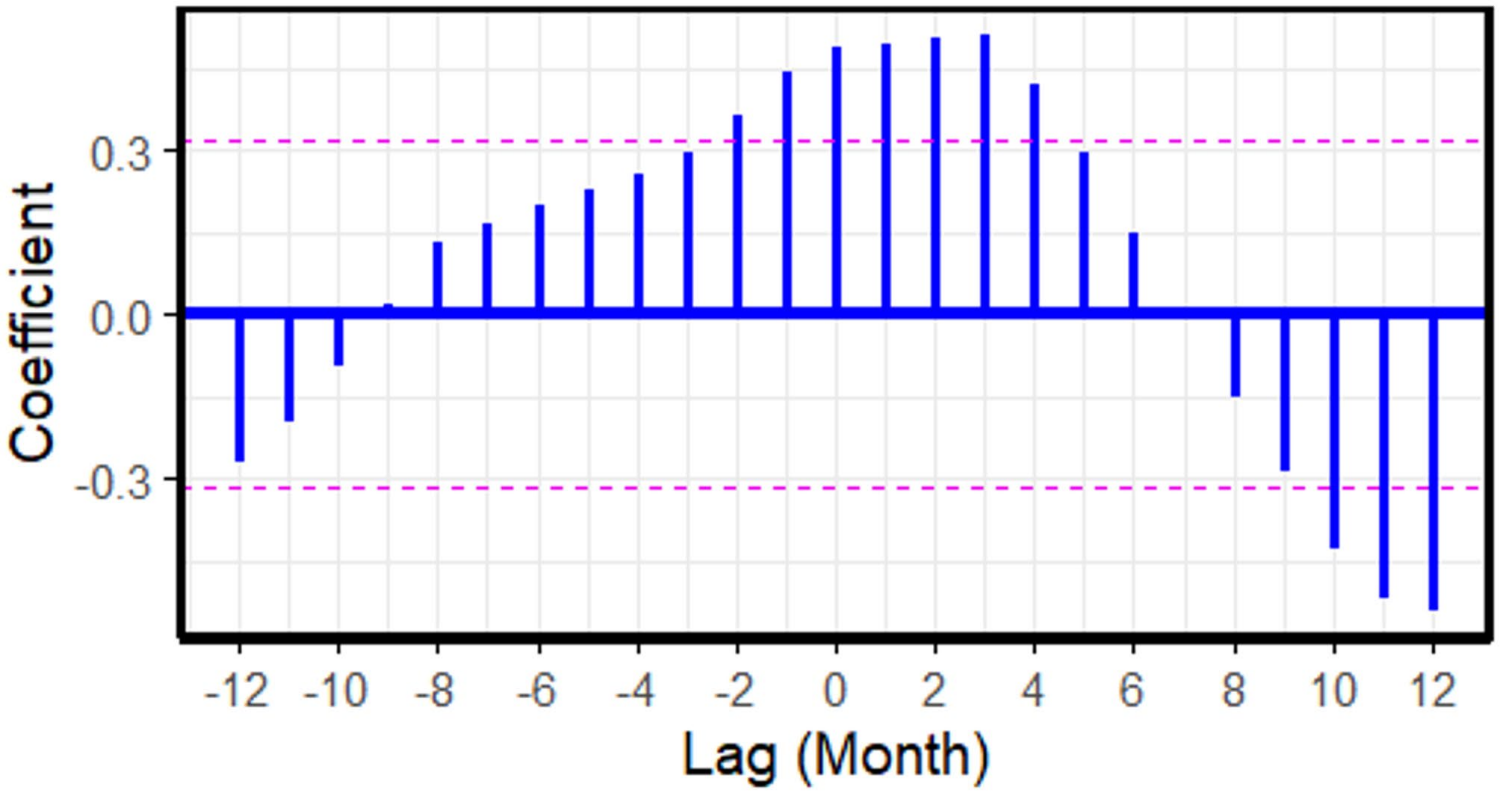
Cross-correlation coefficients between cattle prices and foot and mouth disease outbreak reports across monthly lags, based on 2021 to 2023 data. Each vertical blue bar represents the correlation at a specific lag, with negative lags indicating that changes in animal prices precede changes in outbreak reports. The dashed horizontal lines represent the 95% confidence interval. Bars extending beyond these lines indicate statistically significant correlations.

**Fig. 3:**
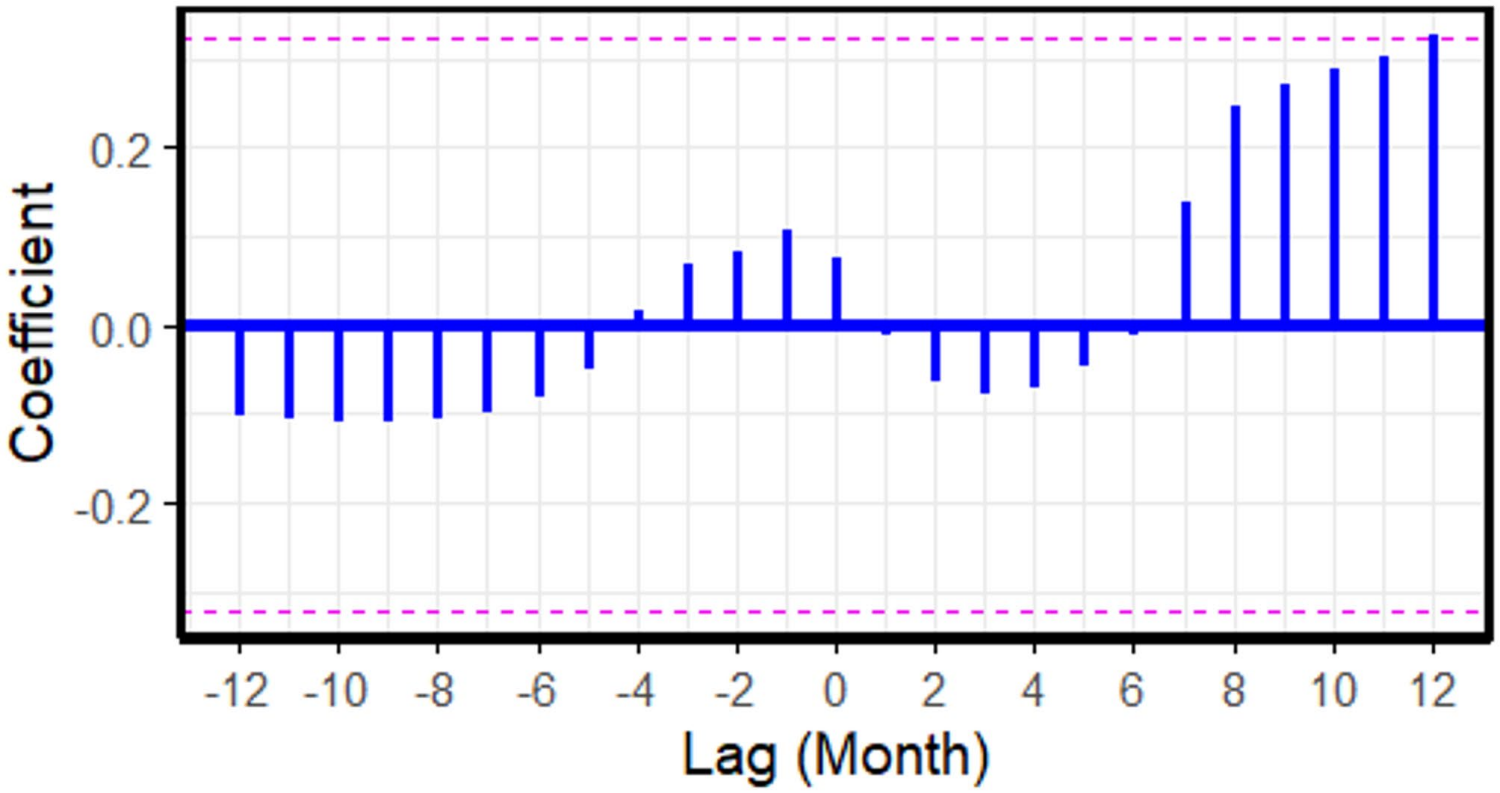
Cross-correlation coefficients between cattle prices and lumpy skin disease outbreak reports across monthly lags, based on 2021 to 2023 data. Each vertical blue bar represents the correlation at a specific lag. The dashed horizontal lines represent the 95% confidence interval. Bars extending beyond these lines indicate statistically significant correlations.

**Fig. 4:**
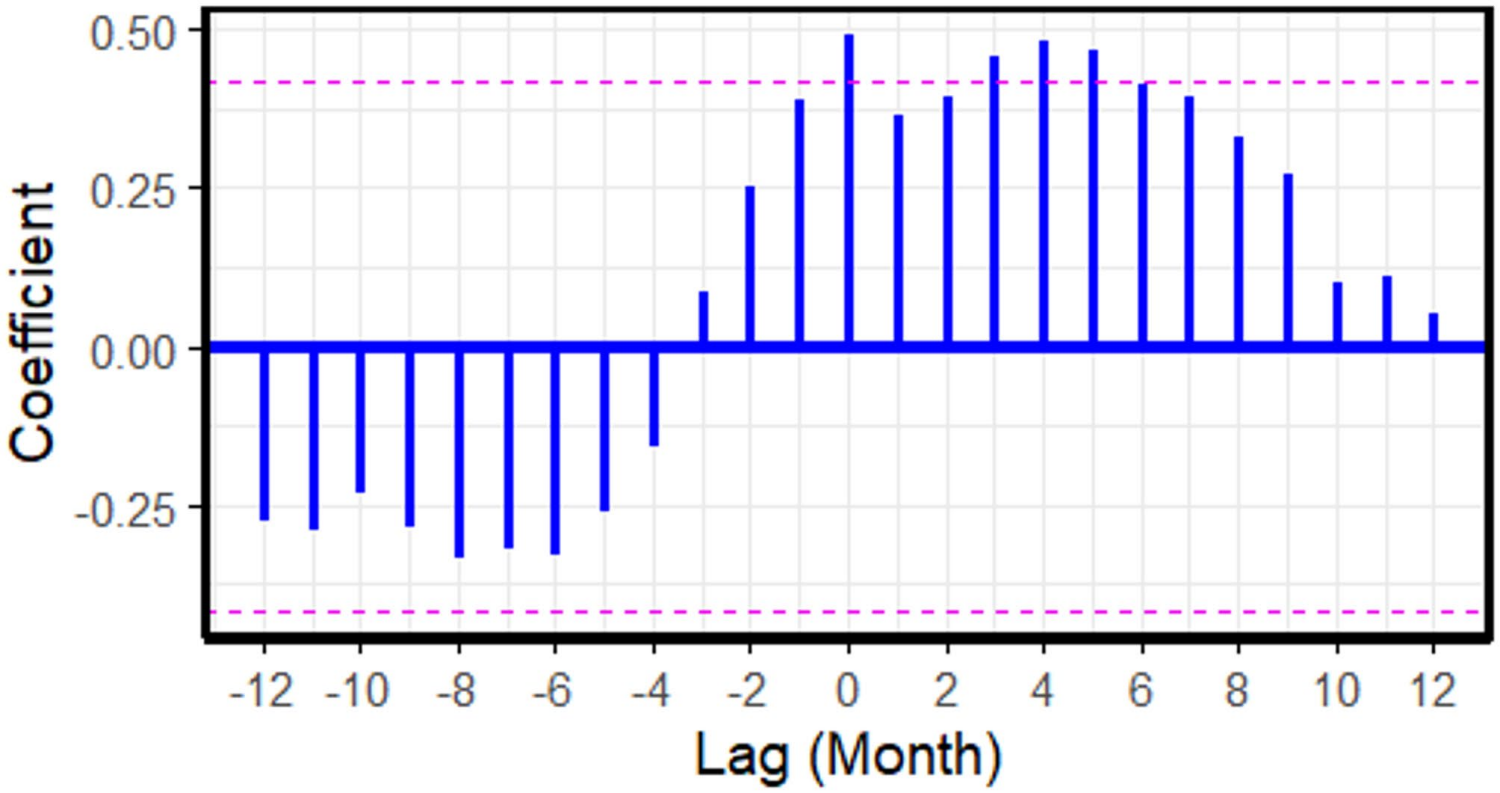
Cross-correlation coefficients between farm-gate pig prices and African swine fever outbreak reports across monthly lags, based on 2021 to 2023 data. Each vertical blue bar represents the correlation at a specific lag. The dashed horizontal lines represent the 95% confidence interval. Bars extending beyond these lines indicate statistically significant correlations.

For FMD, the cross-correlation analysis at negative lags revealed that increases in cattle prices during the preceding 1–2 months ($\:lag=-1$ and $\:lag=-2$) were correlated with a subsequent rise in FMD outbreak reports (Fig. 2; Table 2). In Model 2, no significant lags were identified between cattle prices and LSD outbreak reports (Fig. 3). For ASF (Model 3), cross-correlations between farm-gate pig prices and ASF outbreak reports were observed at positive lags (Fig. 4). Notably, significant cross-correlations between cattle prices and FMD outbreak reports were also observed at positive lags (Fig. 2).

**Table 2:** Cross-correlation results between cattle prices and foot and mouth disease outbreak reports across varying time lags.

Lag*	-12	-11	-10	-9	-8	-7	-6	-5	-4
Coefficient	-0.27	-0.195	-0.093	0.018	0.129	0.166	0.197	0.226	0.256
Lag	**-3**	**-2**	**-1**	**0**	**1**	**2**	**3**	**4**	**5**
Coefficient	0.295	0.362	0.444	0.49	0.493	0.508	0.509	0.423	0.294
Lag	**6**	**7**	**8**	**9**	**10**	**11**	**12**		
Coefficient	0.149	0.001	-0.151	-0.286	-0.427	-0.519	-0.542		

*Lag refers to monthly lag

**Fig. 5:**
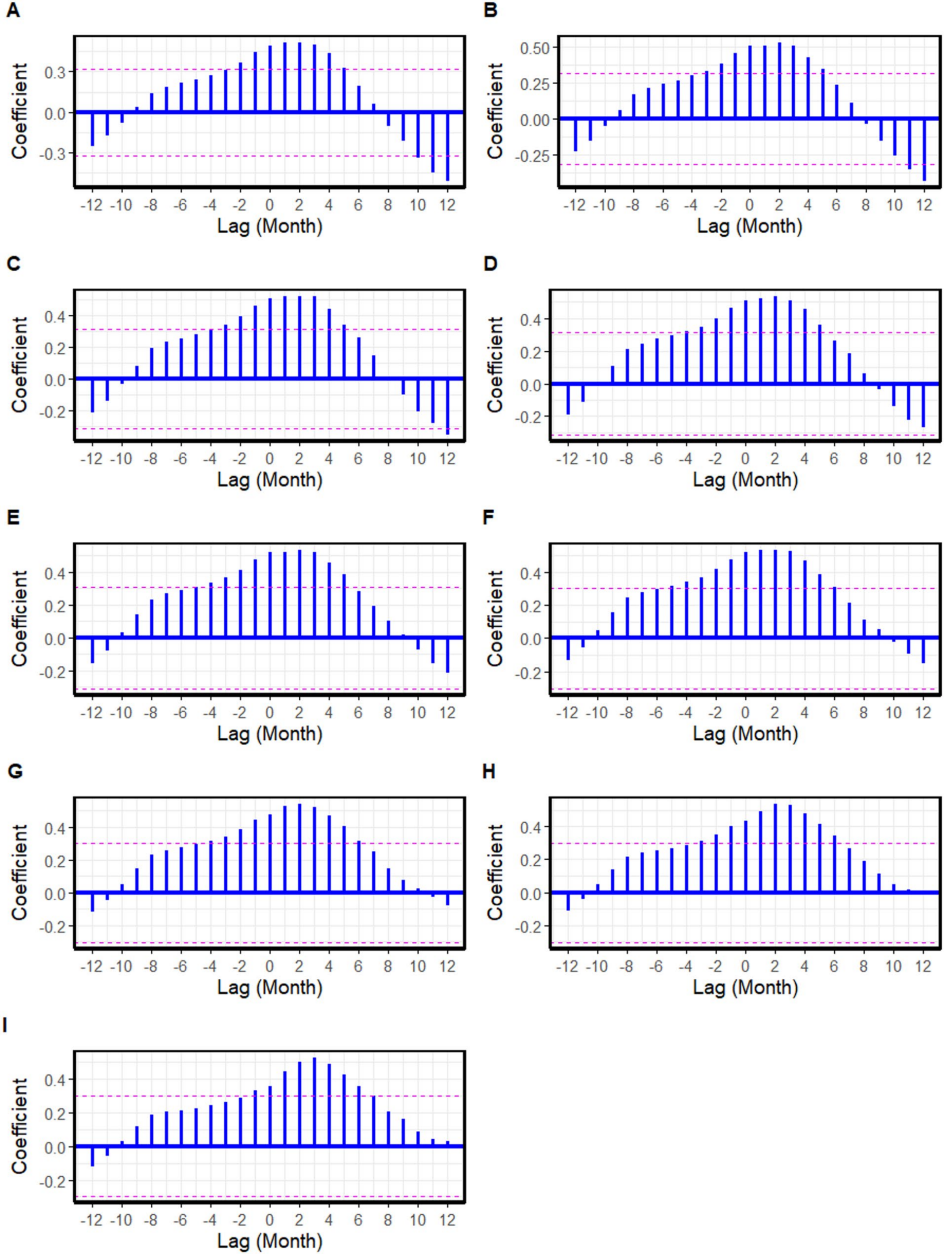
Cross-correlation between cattle prices and foot and mouth disease outbreak reports in the extended dataset from January to September 2024, presented sequentially as letters A to I, respectively. Each vertical blue bar represents the correlation at a specific lag. The dashed horizontal lines represent the 95% confidence interval. Bars extending beyond these lines indicate statistically significant correlations.

With extended data, the cross-correlation between cattle prices and FMD outbreak reports still existed at negative lags as illustrated in Fig. 5. In contrast, no significant cross-correlation existed between farm-gate pig prices and ASF outbreaks.

#### Cattle prices and the number of FMD affected cattle (Model 4)

**Fig. 6:**
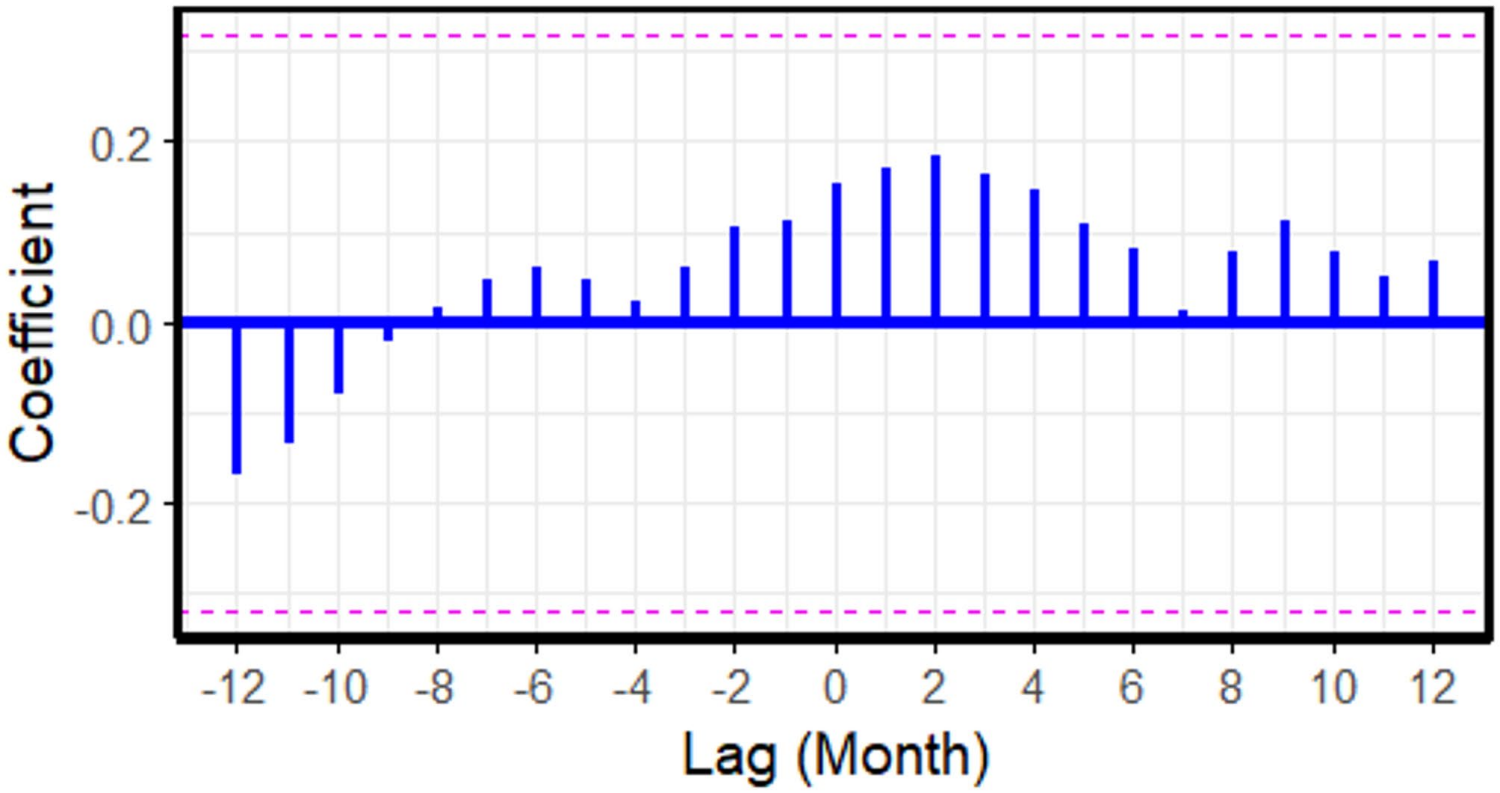
Cross-correlation coefficients between cattle prices and the number of foot and mouth disease affected cattle across monthly lags, based on 2021 to 2023 data. Each vertical blue bar represents the correlation at a specific lag. The dashed horizontal lines represent the 95% confidence interval. Bars extending beyond these lines indicate statistically significant correlations.

According to Fig. 6, no cross-correlation exists between cattle prices and the number of FMD affected cattle.

#### Cattle movement reports and FMD outbreak reports (Model 5)

**Fig. 7:**
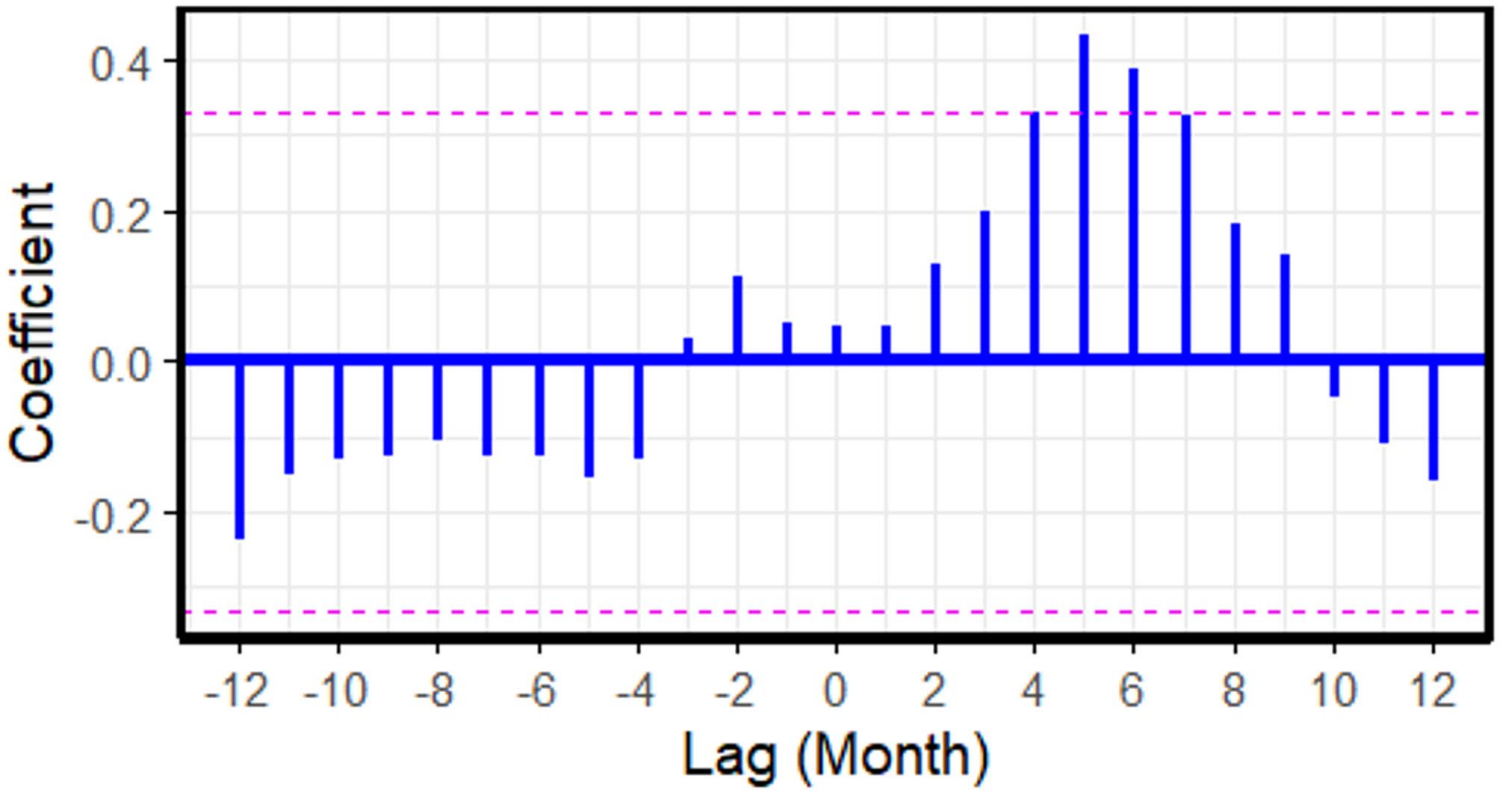
Cross-correlation coefficients between cattle movement reports and foot and mouth disease outbreak reports across monthly lags, based on 2021 to 2023 data. Each vertical blue bar represents the correlation at a specific lag. The dashed horizontal lines represent the 95% confidence interval. Bars extending beyond these lines indicate statistically significant correlations

The significant positive lag (Fig. 7) suggests that an increase in the number of FMD outbreaks ($\:x$ - variable) is followed by a rise in cattle movement reports ($\:y$ -variable) four to seven months later.

### Changes in prices and outbreak reports based on GLM

The seasonally adjusted model outperformed the baseline model, as indicated by a lower AIC value (175.04 vs. 183.50), suggesting a better overall fit to the data. Diagnostic checks, including assessments of residual normality, homoscedasticity, and influential observations, revealed no violations of model assumptions. Therefore, all subsequent interpretations were based on the seasonally adjusted model. Notably, the results from the baseline model are presented in Supplementary Material 1.

The results from the seasonally adjusted model are presented in Table 3. The coefficient for cattle price was estimated at 0.000638 which corresponds to $\:{e}^{\beta\:}={e}^{0.000638}\approx\:1.000638$. This indicates that for each unit increase in cattle price, the expected number of FMD outbreak reports increases by 0.0638%, or 1.000638 folds. When interpreted in the context of a 1,000 THB increase in cattle price, $\:{e}^{\beta\:}={e}^{\left(0.000638*1000\right)}\approx\:1.89$, this indicates that the expected number of outbreak reports is approximately 1.89-fold higher, corresponding to an 89% increase. The 95% confidence interval for this estimate ranged from 1.48, to 2.42.

**Table 3:** Association between cattle prices and foot and mouth disease outbreak reports estimated using a generalized linear model with a negative binomial distribution, adjusted for calendar month

Variable	Estimate		Std. Error		z value		Pr(>|z|)
Intercept	-20.0095		4.324		-4.628		0.00000368
Price_lag*	0.000638		0.000126		5.073		0.000000392
Month							
January	Reference class						
February	-1.225		0.7766		-1.578		0.1146
March	-1.146		0.685		-1.673		0.0944
April	-1.071		0.6805		-1.574		0.1155
May	-1.116		0.6899		-1.617		0.1059
June	-38.86		38,750,000		0.00		1
July	-0.362		0.6382		-0.567		0.5706
August	0.0987		0.6267		0.157		0.8749
September	0.8822		0.6055		1.457		0.1451
October	0.6879		0.612		1.124		0.261
November	0.2767		0.6294		0.44		0.6603
December	0.4147		0.6311		0.657		0.5111

*Price_lag refers to monthly lag of animal price at lag2

## Discussion

Outbreaks of transboundary animal diseases significantly affect animal trading and prices. It is hypothesized that changes in animal prices relate to a shift in outbreak reports. This study explored the potential of using animal prices to predict changes in TAD outbreak reports by analyzing publicly available online data through a cross-correlation model.

This study identified a cross-correlation between cattle prices and the number of FMD outbreaks in the primary dataset, suggesting that an increase in cattle prices during a given month precedes a rise in FMD outbreaks one to two months later. To assess the consistency of this finding, extended data were incrementally incorporated into the analysis. The cross-correlation pattern generally persisted over time, providing preliminary support for the stability of the observed association. As for its implications, the observed cross-correlation at negative lags suggests the potential for using rising cattle prices as an early warning signal for an increase in FMD outbreaks. For instance, an increase in cattle prices in a given month suggests that authorities should closely monitor FMD outbreaks in the subsequent one to two months. There are several plausible explanations for the observed increase in livestock prices preceding FMD outbreaks. One possible explanation is that rising prices may incentivize increased animal movement and trade, thereby elevating the risk of disease transmission and subsequent outbreaks. Another possible explanation is the time lag between the onset of an outbreak and its formal documentation. These delays are common and can be attributed to the sequential processes of outbreak detection, field investigation, laboratory confirmation, and administrative reporting. For example, an outbreak that begins in a given month may not be officially documented until two or three months later. In the meantime, market supply may decline if animals are withheld from sale due to illness or precautionary measures, causing prices to rise and creating the appearance of price increases preceding outbreak reports. Additionally, some outbreaks may initially go undetected, resulting in a temporary reduction in livestock supply, affecting market dynamics. These outbreaks may only be recognized and reported at a later stage, further reinforcing the observed gap between price signals and official outbreak data.

In contrast to FMD, no cross-correlation was observed between cattle prices and LSD outbreak reports (Model 2). This difference is likely attributed to the distinct patterns observed in the outbreak reports of the two diseases. Notably, LSD outbreak reports exhibit a sharp rise to a peak, followed by a slightly sharp decline and consistently low levels of incidence thereafter. Meanwhile, FMD outbreak reports exhibit regular fluctuations over time, as observed in this study, and are likely to follow a seasonal pattern, as reported in previous studies [25, 35]. As a result, the limited variation of LSD outbreak reports in the data may explain why cross-correlation was not detected. Furthermore, ASF displayed a distinct pattern in its relationship with animal prices, differing from both FMD and LSD. For ASF, analysis using the primary dataset (January 2021 to December 2023) revealed significant associations at positive lags between farm-gate pig prices and ASF outbreak reports, suggesting that changes in pig prices were more likely to follow, rather than precede, shifts in outbreak patterns. Moreover, no significant cross-correlations were observed when using the extended dataset (January 2024 to September 2024). Collectively, these findings do not support the use of farm-gate pig prices as an early indicator of ASF outbreak activity in this context. Consequently, the findings from this study do not support the use of animal prices as a predictive signal for an impending increase in ASF outbreaks. Notably, from an epidemiological standpoint, the contrasting findings of negative cross-correlations for FMD and positive cross-correlations for ASF may be attributed to differences in outbreak trends, patterns, and magnitudes between the diseases. These differences may be influenced by various factors, including farmers’ reporting behaviors, as well as response strategies, control measures, and the implementation of disease surveillance systems by livestock authorities.

Based on the findings of this study, using animal prices as an indicator for changes in outbreak reports appears to be more effective for FMD. This is likely because FMD is endemic in Thailand, with outbreak reports occurring consistently throughout the year [25, 36]. Such stability allows for a more predictable and sustained relationship between price trends and outbreak patterns, making FMD a more suitable candidate for price-based surveillance tools. However, it is important to note that ASF outbreak data from other countries potentially reflecting different outbreak dynamics and reporting practices, could yield different results and should be explored in future studies.

In addition to investigating the potential use of cattle prices as a signal for FMD outbreaks, this study also evaluated the use of animal movement data as an alternative signaling tool. However, the findings do not suggest that animal movement data can be used as a practical pre-signal for predicting FMD outbreaks. Instead, the analysis revealed that FMD outbreaks were correlated with subsequent increases in animal movement reports. The analysis revealed a positive correlation, suggesting that increases in cattle movement reports (designated as the 𝑥-variable) were observed four to seven months after increases in FMD outbreak reports (𝑦 variable). This temporal association may also be expressed in reverse: when the number of FMD outbreak reports (𝑥-variable) increases in a given month, a corresponding rise in cattle movement reports (𝑦-variable) is generally observed four to seven months later. This finding aligns with a common scenario in Thailand, where animal movement is typically restricted for at least one month following the last reported FMD case in an outbreak area [37]. In this practice, the duration of these restrictions may vary depending on the severity of the outbreak. For instance, if the outbreak lasts for three months, animal movement restrictions will typically extend to four months. After the restriction period ends, cattle trading activities resume, and animal movements are likely higher compared to the restriction period.

Interestingly, no significant cross-correlation was identified between cattle prices and the number of cattle affected by FMD. While it might be expected that a higher number of affected cattle during outbreaks would lead to price increases, this relationship was not observed. This lack of correlation could be attributed to the substantial variability in the number of affected cattle across different outbreaks. Moreover, changes in animal prices may be influenced by factors beyond the number of affected cattle. For example, restrictions on animal movement during outbreaks can significantly influence prices by reducing the number of animals available in the market. This occurs because cattle within outbreak and surveillance areas are subject to movement controls [23]. Additionally, factors such as market availability [38, 39] and policy interventions [40] likely contribute to these patterns and should be explored in future studies.

Based on the findings of this study, authorities can interpret an increase in animal prices as a potential early warning signal for an impending rise in FMD outbreaks. In response, veterinary services can enhance surveillance efforts in the affected region, allocate resources to monitor cattle health, and implement preparedness measures to manage a potential outbreak. Nevertheless, as demonstrated in this study, using animal prices as a signaling tool to predict changes in outbreak reports presents several challenges. For example, government interventions, such as price stabilization policies for pigs, can disrupt the expected cross-correlation between prices and outbreaks. Additionally, sudden spikes in outbreak reports may occur without prior signals derived from animal prices. These complexities emphasize the need to integrate multiple data sources and consider contextual factors when refining predictive models. Furthermore, data availability plays an essential role in establishing animal prices as a tool for predicting changes in outbreak reports [22]. The present study has been able to explore the potential of this approach because animal price data in Thailand have been consistently available and regularly updated on online platforms. Expanding this methodology to address transboundary diseases across broader regions, such as Asia or Southeast Asia, will require the availability of comparable data from multiple countries [22, 41]. Additionally, ensuring the consistency and quality of data across regions will be essential for effectively applying cross-correlation methods to support disease surveillance and management efforts.

This study has several limitations. First, the available animal price data were not disaggregated by region (e.g., northern or central Thailand), making regional-level analysis unfeasible. Additionally, as the primary objective was to explore temporal relationships rather than to establish causal inference using real-world surveillance data, cross-correlation analysis was employed to assess potential associations in time series data exhibiting non-constant behavior. It is also important to acknowledge that the observed associations between animal prices and outbreak reports may be spurious, potentially influenced by confounding factors such as seasonality. Accordingly, the findings should be interpreted as exploratory and indicative of temporal associations, rather than as evidence of causality. Moreover, since the data used in this study were limited to Thailand, the results should not be generalized to other countries. This geographic constraint also prevented the assessment of model robustness across different settings. Given that the dataset covered only a three-to-four-year period, the time series was relatively short. To strengthen the reliability and applicability of the findings, future studies should incorporate longer time series and include data from multiple countries. Furthermore, the under-reporting of animal disease outbreaks [41–44] may have affected the quality of the outbreak data and introduced potential bias into the cross-correlation analysis. Finally, as this was a pilot investigation, the study was limited to a single analysis with a univariable approach. Future research may consider employing more advanced statistical techniques or comprehensive multivariable frameworks to enhance the predictive value of animal price data for anticipating increases in outbreak reports.

## Conclusion

This study demonstrates the potential of leveraging animal prices and movement data as early signal tools for FMD, LSD, and ASF outbreaks. A cross-correlation at negative lags between cattle prices and FMD outbreak reports suggests that price increases may serve as early warning signals for a subsequent rise in FMD outbreaks. In contrast, no significant cross-correlations were observed for LSD or ASF, likely due to differences in their outbreak dynamics. The findings from this pilot study provide a valuable basis for using animal prices as an additional tool to support decision-makers and veterinary authorities in early warning and surveillance strategies for FMD in Thailand.

## Electronic supplementary material

Below is the link to the electronic supplementary material.

Supplementary Material 1

## Data Availability

The data utilized in this study are publicly available online, as detailed in the manuscript. Animal price data can be accessed at https://dld.go.th and https://www.oae.go.th. Information on animal disease outbreaks is available at https://wahis.woah.org.
