# Supplementary material for: Using online public animal price data as a signal for predicting an increase in animal disease outbreak reports: a pilot study on cross-correlation modeling in Thailand
Source: BMC Vet Res. 2025 Jul 2;21:427. doi: 10.1186/s12917-025-04888-5 (PMC12219664; doi:10.1186/s12917-025-04888-5)
Supplement: Supplementary file 1 — Supplementary Material 1 [file 12917_2025_4888_MOESM1_ESM.docx]

**Table S1.** Association between cattle prices and foot and mouth disease outbreak reports estimated using a generalized linear model with a negative binomial distribution.

| Variable | Estimate | Standard Error | z value | P-value |
| --- | --- | --- | --- | --- |
| Intercept | -12.48 | 6.567 | -1.901 | 0.04 |
| Price | 0.000413 | 0.000193 | 2.136 | 0.03 |
